# Robust Planning for a Patient Treated in Decubitus Position with Proton Pencil Beam Scanning Radiotherapy

**DOI:** 10.7759/cureus.1706

**Published:** 2017-09-20

**Authors:** Shikui Tang, Limin Song, Jared D Sturgeon, Chang Chang

**Affiliations:** 1 Medical Physics, Texas Center for Proton Therapy; 2 Physician, Texas Center for Proton Therapy

**Keywords:** lung tumors, robust planning, proton raiotherapy, pencil beam scanning

## Abstract

A challenging case was reported for a patient treated in decubitus position with proton pencil beam scanning. A regular robust plan with the consideration of the uncertainties of translational alignment and range accuracy cannot ensure the target coverage as revealed in two verification computed tomography (CT) scans during the first week of the treatment. The irreproducibility of daily alignment and anatomical variations in such a position is mainly due to patient’s roll. To mitigate the interfractional effect on the target coverage, a novel robust optimization against the patient’s angular setup uncertainties was implemented to improve the plan quality by introducing two artificial CT image sets by rolling the planning CT three degrees in both clockwise and counter-clockwise directions and adding them into robust optimization scenarios, which was shown to be an effective and simple way to mitigate target dose degradation with respect to interfractional variations. This method can be easily generalized and applied to other situations where angular variations in patient’s setup can introduce large dosimetric effects. It is recommended that angularly robust optimization method should be integrated into the treatment planning system as an option particularly for patient’s treatment subject to large angular variations, such as the one in the decubitus position reported here.

## Introduction

Proton radiotherapy can provide superior dose distribution with its finite penetration depth in tissue and therefore no exit dose beyond its intended range [1]. But the effectiveness of such superior dose distribution is sensitive to various uncertainties, such as the proton range accuracy in different tissue types, setup variations, target motion, and patient anatomical changes throughout the treatment course. These uncertainties can potentially degrade the planned proton dose distribution and are one of the major challenges in the proton therapy [2-4].

Several planning techniques have been developed to account for these uncertainties. One method is the use of a planning target volume (PTV) expanded from the clinical target volume (CTV) with predetermined margins. Like in conventional photon radiotherapy, the margin and coverage goal of PTV is experimentally adopted such that the CTV will remain adequately covered with high probability in the presence of various uncertainties [5]. However, this adopted method from photon radiotherapy has been shown to be insufficient in ensuring plan robustness for proton radiotherapy [6-7]. Plans generated by robust optimization, on the other hand, have demonstrated superior resilience to the aforementioned uncertainties by taking them into account during the plan optimization process [8-10].

Robust optimization aims to find the best solution among all scenarios under consideration, including the combinations of uncertainties from setup, density calibration, patient anatomical variations, etc. As a result, the robustness, i.e. the resilience to uncertainties, of the final plan depends on how representative the chosen scenarios are. Currently available scenarios in a typical treatment planning system (TPS) include the following three categories: (1) setup uncertainty executed by translationally shifting the beam isocenter in the patient’s computed tomography (CT) image, (2) range uncertainty implemented by varying the tissue density calibration from the CT’s Hounsfield Unit (HU) to proton nominal range, and (3) anatomical variations achieved by simultaneously optimizing on multiple phases of a four-dimensional CT (4DCT) image set. However, (to the best of our knowledge) none of the current commercially available TPS provides robust optimization with respect to patient rotation in any direction. Lack of this angularly robust optimization functionality poses a limit to patients who need to be treated in positions that are prone to larger angular variations in their day to day setup. For example, for patients treated with lateral decubitus position, one of the major difficulties for daily setup is the patient’s reproducibility in roll. Even though modern treatment couches with six degrees of freedom can indeed correct the roll up to a certain level, the residual angular difference among the soft tissues, bony structures, and the target can still be present during the treatment. In this report, we showed a challenging case where robust planning in patient’s roll is clinically necessary, and demonstrated an innovative method to implement this angularly robust optimization using a commercially available TPS.

## Case presentation

Patient data

**Figure 1:**
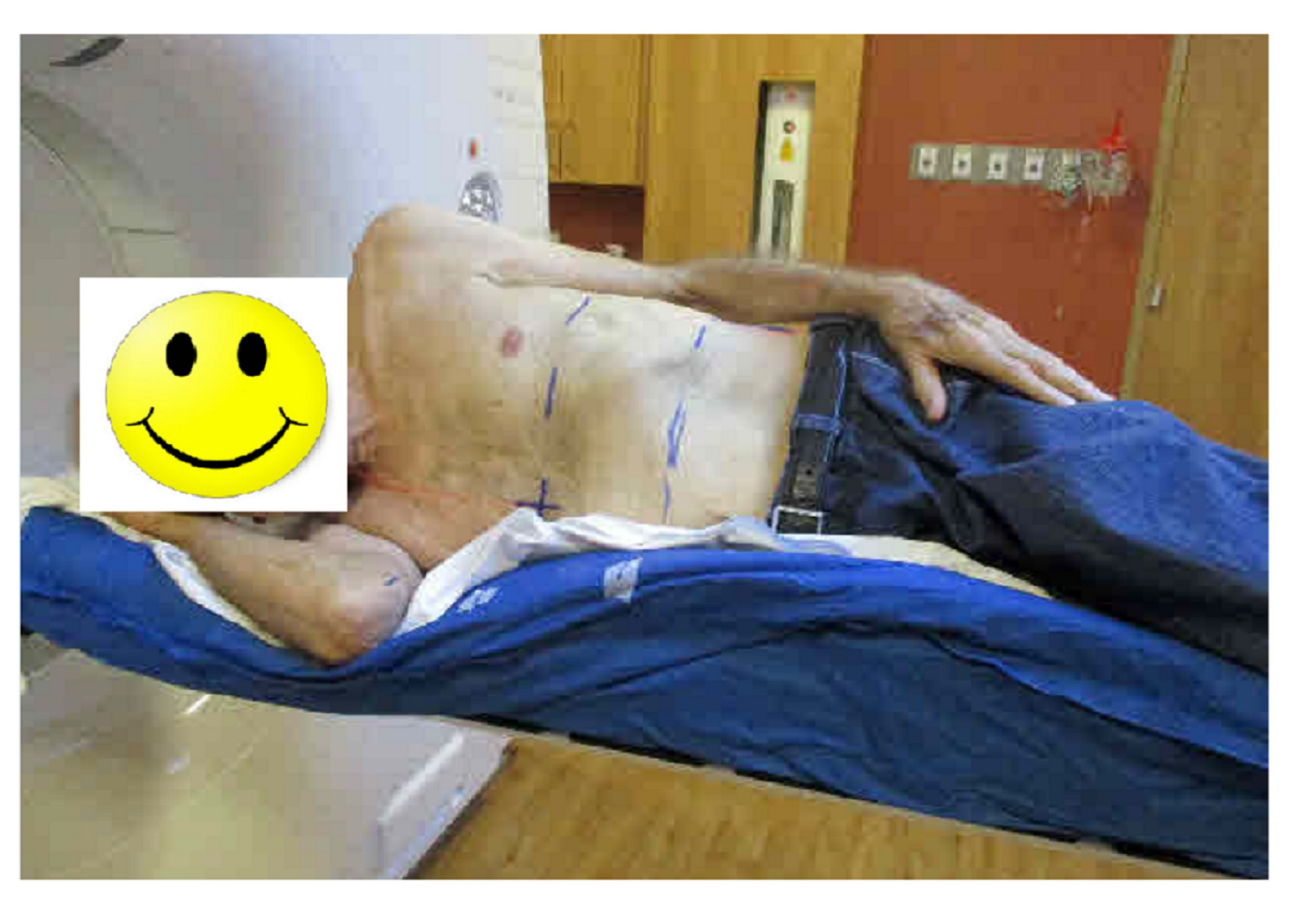
A patient was simulated in a right lateral decubitus position. A whole body Vac-Lok was used for immobilization.

Due to specific medical conditions, a patient can only be simulated in a decubitus. The tumor was in the middle of the left lung. To minimize anatomical deformation of his left chest region during treatment, the patient was simulated in a right lateral decubitus position. A whole body Vac-Lok (Civco Medical Solutions) was used to immobilize the patient (Figure 1).

A 4DCT was performed for this patient to assess the respiration motion. It indicates that the tumor motion is around five millimeter in all directions. The internal gross target volume (iGTV) was obtained by contouring the gross tumor volume (GTV) on individual phases so as to encompass the full motion of the gross tumor in all phases of the 4DCT. The CTV was a five-millimeter uniform expansion from the iGTV. A regular PTV was also created with another five-millimeter expansion from the CTV.

The patient was treated with the guidance of kV orthogonal images and daily cone beam CT (CBCT) for alignment. During the treatment course, two verification CT scans were obtained and registered to the original planning CT using rigid registration. They were used to validate the plan robustness against interfractional variation.

Treatment planning

All plans were generated in the treatment planning system (Raystation 5.0.2® Sweden). An anterior beam and a right posterior beam were adopted for this case where the skin surface is smooth and has the least effect on dose distribution. The TPS supports robust planning with respect to the patient’s translational positioning uncertainty, proton beam range uncertainty, and respiratory motion (4D optimization). For setup robustness, the isocenter is allowed to shift along the anterior-posterior, superior-inferior, and lateral directions by five millimeters. For range uncertainty, a +/-4% perturbation is added to the mass density calibration curve to represent the under-ranged and over-ranged proton penetration depths, respectively. The robustness parameters were applied on the CTV only and their combination results in 21 scenarios being considered during optimization. The nominal plan was generated on the planning CT, which is obtained by averaging the 4DCT data set. The optimization of the nominal plan also takes into account the maximal inhale and maximal exhale phases of the 4DCT in order to take into consideration the effect of respiration motion. The planning goal is to have 95% of the PTV receiving 95% of the prescribed dose of 80 Gy(RBE) and the iGTV is required to receive about 10% higher dose. The total fraction number is 40 with a daily dose of 2 Gy(RBE) for this reported case. Note that the nominal plan includes the robust optimization with respect to both range uncertainty and setup uncertainty except the variation of patient angular alignment.

The robustness optimization with respect to patient rotation, roll or pitch is not directly available in the TPS. An innovative method was developed to artificially include such angular effect during optimization. First, the original planning CT image set was duplicated twice. Then the duplicated CT image sets were rotated three degrees along the superior-inferior axis (i.e., patient’s roll) in both clockwise (CW) and counter-clockwise (CCW) directions, respectively, in order to simulate the patient’s daily setup variations in the decubitus position. Afterwards, the two rolled CT image sets were registered to the original CT image set at the beam isocenter using translational shifts only. Finally, in the TPS robustness settings, these two rolled CT image sets are added to the list of CT images that are to be considered during optimization. In this case, the planning CT, the CT images at selected respiratory phases (i.e., maximal inhale and maximal exhale), and the two rolled CT images are all included. All anatomical scenarios represented by these CT images will be used for dosimetric optimization.

Results

Robustness on Patient Roll

The nominal plan was created with five-millimeter setup uncertainty and ±4% range uncertainty for clinical treatment. The patient was aligned using daily CBCT and orthogonal KV images. The evaluation of daily alignment in the first a few fractions indicated that up to 2-3 degrees of residual roll of the patient’s chest wall would typically remain even after the target and the vertebra were well aligned to the digitally reconstructed radiograph (DRR). Such residual discrepancy in patient’s roll can be mitigated by manipulating the patient’s position several times during initial setup; however, it was not completely eradicated. Moreover, the residual rotation will likely persist throughout the entire course of treatment.

**Figure 2:**
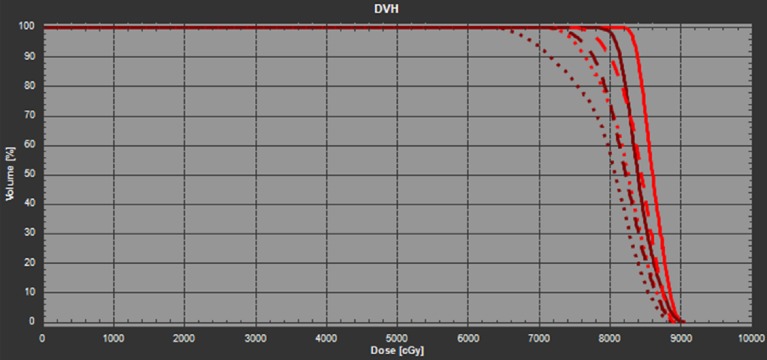
The DVH of the nominal plan in the planning CT (solid line), +3 degree (CW) rotated CT (dashed line), and -3 degree (CCW) rotated CT (dotted line). The red lines denote the iGTV and the dark brown lines denote the CTV. CCW: Counter-clockwise; CT: Computed tomography; CTV: Clinical target volume; CW: Clockwise; DVH: Dose volume histogram; iGTV: internal gross target volume.

To quantitatively evaluate the robustness of the nominal plan against the patient’s residual misalignment in roll, the gantry angle of each of the beam was changed by +/-3 degrees and then the plan was re-calculated without re-optimization. Note that the resultant dosimetric distribution is equivalent to forward calculating of the nominal plan on the rolled CT images duplicated from the original planning CT. The DVHs of iGTV and CTV were shown in Figure 2. The dosimetric variations of iGTV D95 and D50 relative to the corresponding values in the nominal plan are listed in Table 1. As observed, due to the lack of angular robustness in roll during optimization, the target coverage is significantly compromised in the two tested scenarios where the patient is rolled by ±3 degrees. For example, the iGTV D95 will be reduced by 10.5% and CTV D95 reduced by 14.5% if the patient is to be rolled counterclockwise by three degrees. The coverage for iGTV D95 and CTV D95 can also be reduced by 5.9% and 5.8%, respectively, if the patient rolls three degrees to the opposite direction.

**Table 1:** The dosimetric variation of the nominal plan and RR plan under scenarios of rotated patient planning image CT (-3 and +3 degree along the superior-inferior axis) and verification CT scan (VFCT1 and VFCT2). The $\Delta$​​​​​​​ is defined as the percentage change of each scenario relative to the plan on the planning CT, which is less affected by the slight dose difference between the nominal plan and RR plan and helps to compare the robustness of the two plans. CCW: Counter-clockwise; CT: Computed tomography; CTV: Clinical target volume; CW: Clockwise; iGTV: internal gross target volume; RR: Roll robust; VFCT: Verification CT.

	$\Delta$iGTV D95	$\Delta$iGTV D50	$\Delta$CTV D95	$\Delta$​​​​​​​​​​​​​​CTV D50
The nominal plan				
-3 degree (CCW)	-10.1%	-4.0%	-14.5%	-3.8%
+3 degree (CW)	-5.9%	-1.8%	-5.8%	-2.1%
VFCT1	-20.2%	-8.9%	-25.6%	-9.9%
VFCT2	-9.2%	-2.0%	-13.8%	-1.8%
RR plan				
-3 degree (CCW)	-1.2%	-0.8%	-1.2%	-0.2%
+3 degree (CW)	-3.3%	-1.4%	-3.2%	-1.0%
VFCT1	-5.4%	-2.4%	-7.3%	-1.8%
VFCT2	-2.7%	-0.3%	-4.7%	0.0%

**Figure 3:**
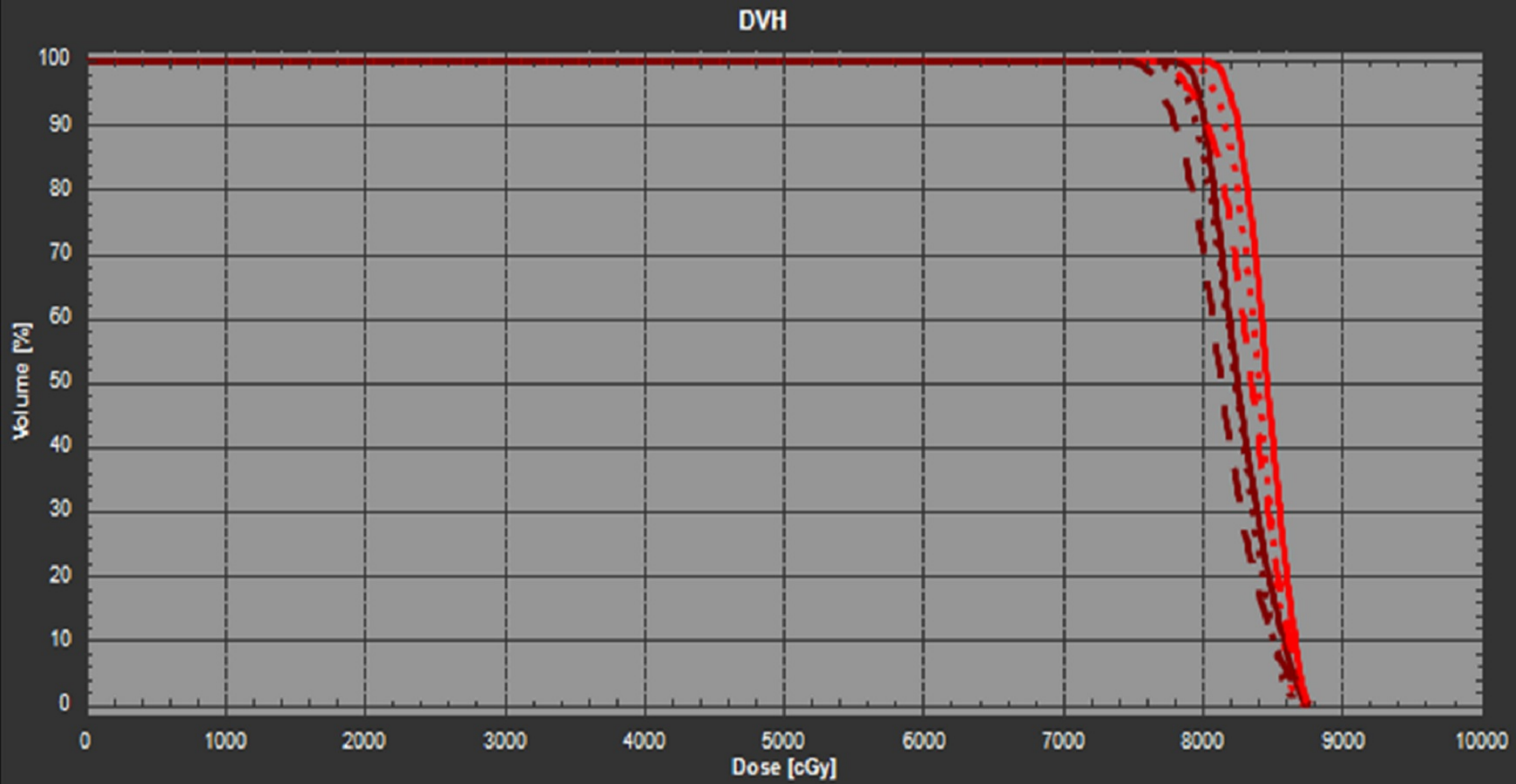
The DVH of the RR plan in the planning CT (solid line), +3 degree (CW) rotated CT (dashed line), and -3 degree (CCW) rotated CT (dotted line). The red lines denote the iGTV and the dark brown lines denote the CTV. CCW: Counter-clockwise; CT: Computed tomography; CTV: Clinical target volume; CW: Clockwise; DVH: Dose volume histogram; iGTV: internal gross target volume; RR: Roll robust.

To increase the robustness against patient angular uncertainty during treatment, a revised plan was generated which included its robust optimization in those two additional duplicated CT images from the planning CT. The new plan was referred as the roll robust (RR) plan hereafter. The corresponding dose matrices and DVH plots of iGTV and CTV for the ±3 degree roll scenarios were shown in Table 1 and Figure 3. By default, the RR plan should ensure the target coverage with respect to patient’s roll uncertainty. The three-degree scenario received the most improvement in the robust optimization. Compared to the nominal plan, the RR plan has significantly less reduction in target coverage (3.3% in the worst case) given the same ±3 degrees of patient’s roll. The iGTV coverage is therefore much better preserved in the RR plan. As a result, the patient treatment was switched to the new RR plan immediately after it became available.

Robustness on Verification CT Scans

**Figure 4:**
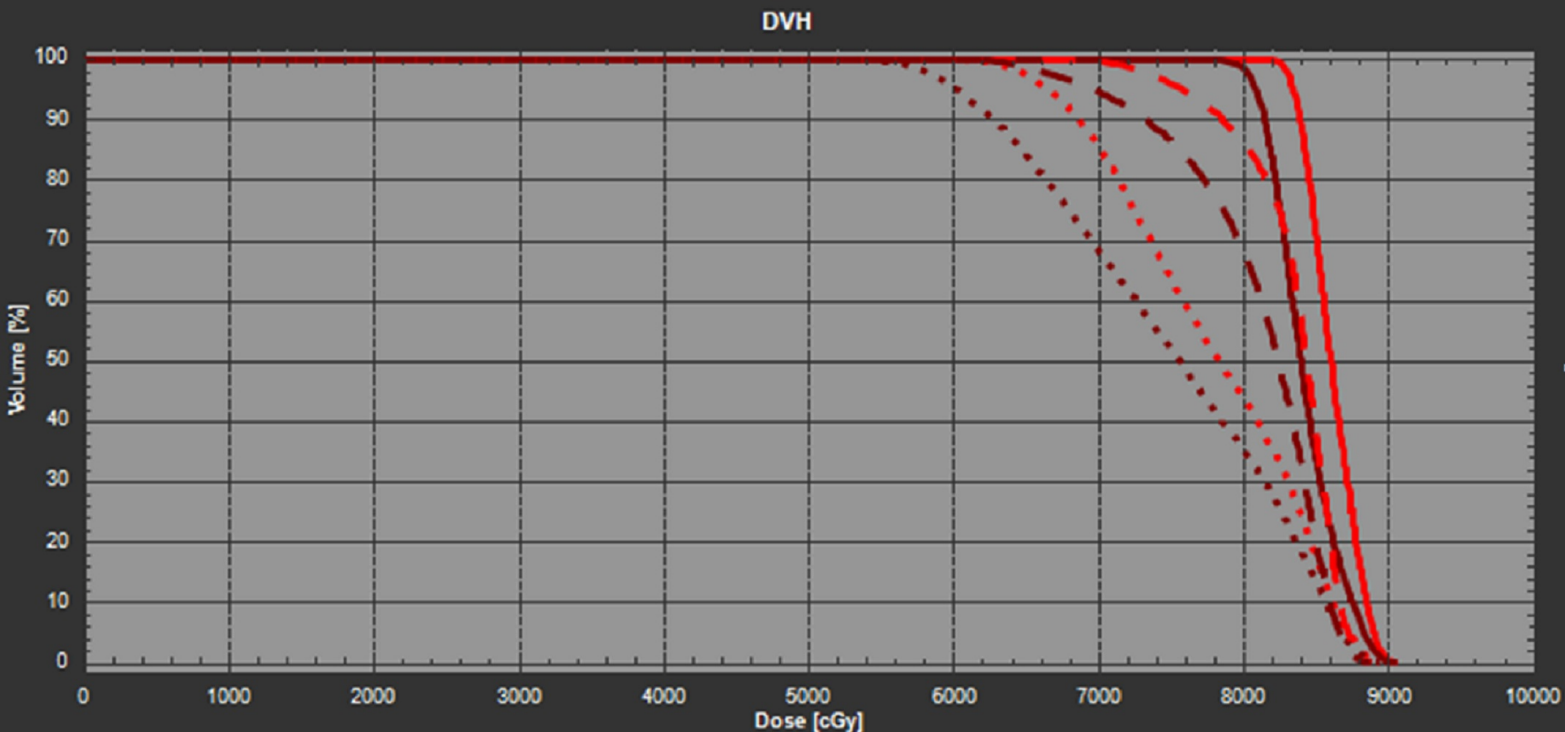
The DVH of the nominal plan in the planning CT (solid line), VFCT1 (dotted line), and VFCT2 (dashed line). The red lines denote the iGTV and the dark brown lines denote the CTV. CT: Computed tomography; CTV: Clinical target volume; DVH: Dose volume histogram; iGTV: internal gross target volume; VFCT: Verification CT.

**Figure 5:**
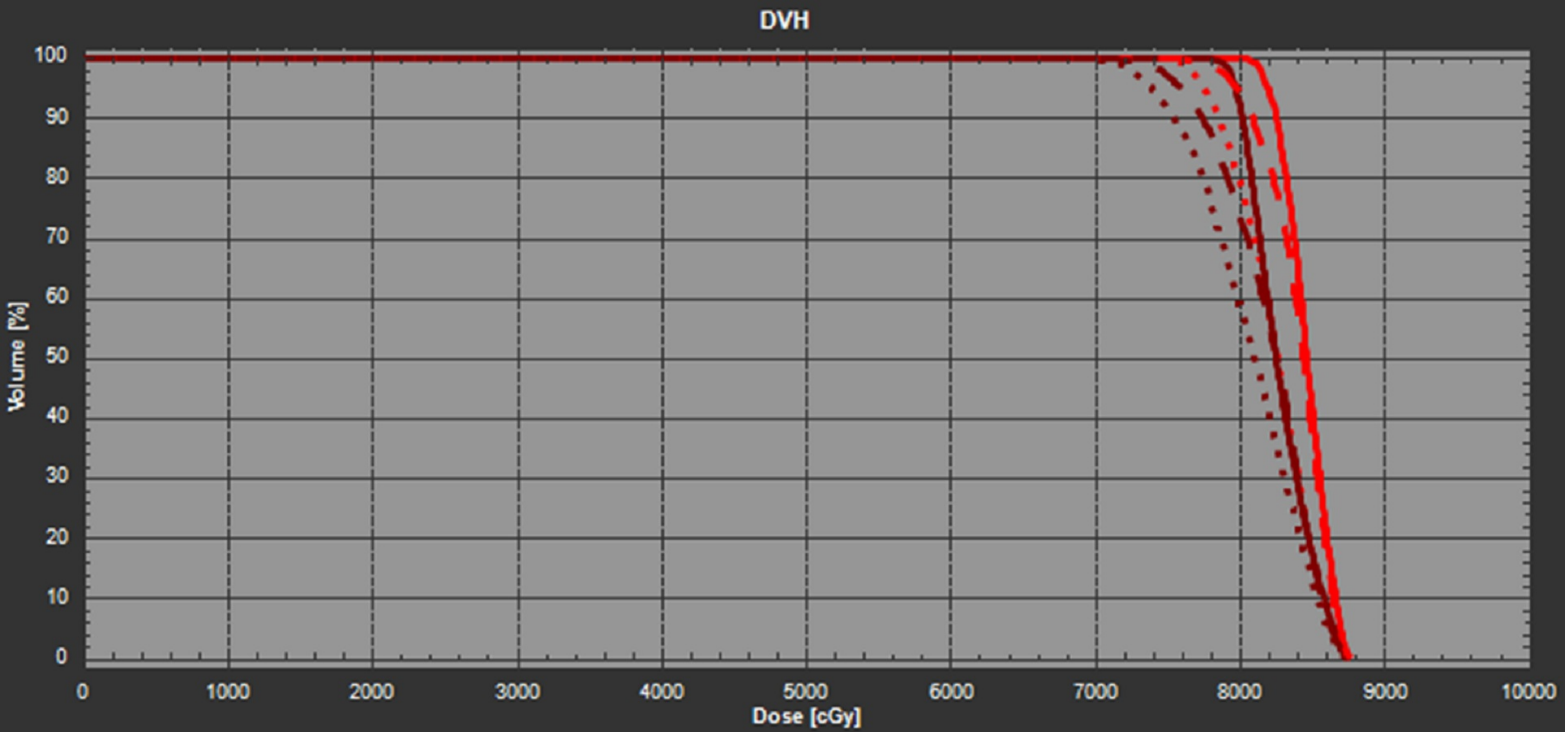
The DVH of the RR plan in the planning CT (solid line), VFCT1 (dotted line), and VFCT2 (dashed line). The red lines denote the iGTV and the dark brown lines denote the CTV. CT: Computed tomography; CTV: Clinical target volume; DVH: Dose volume histogram; iGTV: internal gross target volume; RR: Roll robust; VFCT: Verification CT.

Two verification CT (VFCT) scans were performed during the first week of treatment to check the patient’s daily setup reproducibility, and to evaluate the interfractional dose variations. Both the nominal plan and the RR plan were forward calculated on these VFCT images. The DVHs of iGTV and CTV for the nominal plan and the RR plan were shown in Figure 4 and Figure 5, respectively. Results of their dosimetric comparison are listed in Table 1. For the nominal plan, the iGTV D95 was reduced by 20.2% in VFCT1 and 9.2% in VFCT2; the CTV D95 was reduced by 25.6% in VFCT1 and about 13.8% in VFCT2. In clinical practice, such dramatic coverage degradation typically requires immediate plan revision. For the RR plan, the corresponding target coverages for iGTV D95 were reduced by 5.4% on VFCT1 and 2.7% for VFCT2. It is worthwhile to mention that although the RR plan was generated on the original planning CT and two artificially rolled planning CT, it was robust against interfractional variations, which include both patient internal anatomy changes over time as well as interfractional setup uncertainties in both translational and angular directions.

## Discussion

A challenging case was reported where the patient was treated in a decubitus position. Compared to the commonly employed supine or prone setups, it is more difficult to align patients in the decubitus position primarily due to the irreproducibility of the patient’s roll. In addition, the interfractional variation in roll can cause a patient to have internal anatomic variations in tumor position relative to adjacent bony structures and the outline of chest wall. From this patient’s daily CBCT alignment, variations in the shape of the chest wall were observed after aligning to the target and the overall anatomy. Such chest wall deformation resembled two to three degrees of roll in the patient by just matching the shape of chest wall. The variation of the chest wall shape introduces range uncertainty that was not included in the regular robust setting such as the isocenter shift and density perturbation [8-10]. Although the nominal plan’s robust optimization indeed accounted for translational setup uncertainty, range uncertainty, as well as respiration motion using 4DCT image sets, target coverage of the plan was not ensured on the two VFCTs. The large degradation in target coverage seen in the two VFCTs indicated that for patients in the lateral decubitus position, daily variations in setup reproducibility can cause significant interfractional target dose degradation. It is also noted that the residual roll between the target and the chest wall is the likely culprit. The clinical plan therefore needs to be carefully designed to mitigate this interfractional effect in order to avoid underdosing of the target.

**Figure 6:**
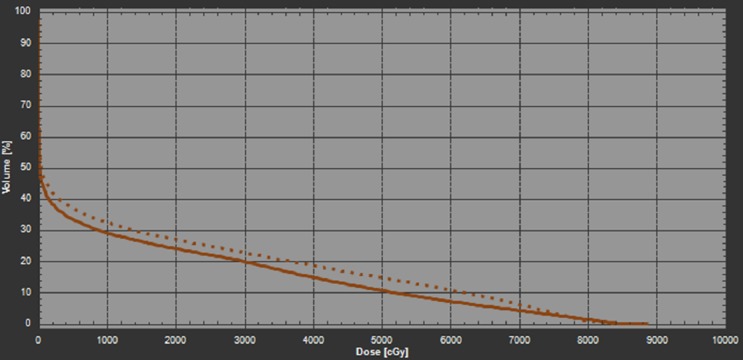
The DVH of the left lung of the nominal plan (solid line) and the RR plan (dotted line). DVH: Dose volume histogram; RR: Roll robust.

Robust optimization against the patient’s angular setup uncertainties is crucial to improve the plan quality for this case. Our approach of creating angularly shifted, i.e., rolled in this case, extra CT images from the original planning CT, and adding these additional CT images into the robust optimization scenarios is shown to be an effective way to mitigate target dose degradation. Our results show that the RR plan created in this angularly robust fashion can significantly increase the plan robustness against the interfractional variation mainly resulted from the patient’s roll. Indeed, the RR plan maintained the target coverage on the two VFCTs. This method can be generalized and applied to other situations where angular variations in the patient’s setup can introduce large dosimetric effects. For example, this angularly robust optimization method is needed for beams traversing through regions that have a large slope in anatomical heterogeneity or external contour. Note that the RR plan slightly increased the dose of the left lung (Figure 6) to gain the angular robustness. Ideally, adaptive radiotherapy should be used if the patient's anatomical variation is consistent with respect to CT simulation during the course of the treatment in order to reduce the normal lung dose.

**Figure 7:**
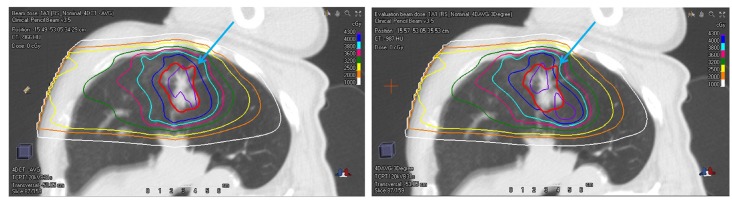
An individual beam dose distribution of the nominal plan on the planning CT (left) and three-degree counter clock-wisely rotated planning CT (right). The thick red line denotes the iGTV. CT: Computed tomography; iGTV: internal gross target volume.

In principle, it is possible to increase the margin of the PTV to ensure target coverage if one can quantify the interfractional variation. However, in practice, it is difficult to implement such a margin for angular setup uncertainties. Indeed, given a fixed angular setup error, water-equivalent thickness (WET) variation along the path of each pencil beamlet can be different, thus a uniform expansion for the margin of the PTV may not be reasonable. As shown in Figure 7, the isodose lines are distorted when the nominal plan was forward calculated on a rolled CT image set, as indicated by the cyan arrow. The relative position between the target and other tissues may also vary at each treatment fraction, which could also change the WET of the beam path and therefore degrade the target coverage. Due to the scarcity of experience in decubitus patient positioning in proton radiotherapy, there is not enough experience and consensus in setting a safe angular margin for the PTV.

An alternative method equivalent or possibly more effective to our approach is to scan the patient multiple times during CT simulation with off-table breaks in-between. Instead of using the manually generated images, all of these CT images are realistic simulations of interfractional setup variations and can be included in the robust optimization. For our case, this is analogous to taking the VFCTs ahead of time and incorporating them into the planning process. Of course, during the treatment course other VFCTs may still be needed for plan validation purposes such as the dosimetric effect of patient weight loss. Note that the patient will receive more CT imaging dose in this approach.

## Conclusions

Our approach of angularly robust optimization shares the same idea as robust optimization using 4DCT image sets. The *RR *plan incorporated angular perturbations into the current available TPS and enforced the robustness criteria on all CT datasets during optimization, which helps to ensure the target coverage with respect to interfractional variations. In fact, this angularly robust optimization can be easily implemented in TPS by simply varying the beam angles (as well as the table angles). It is recommended that this functionality be integrated into the TPS as an option for patient treatment subject to large angular variations, such as the one in the decubitus position reported here.
